# Immunogenicity Risk Assessment for Multi-specific Therapeutics

**DOI:** 10.1208/s12248-021-00642-5

**Published:** 2021-11-05

**Authors:** Mark A. Kroenke, Mark N. Milton, Seema Kumar, Eris Bame, Joleen T. White

**Affiliations:** 1 Amgen Inc, Thousand Oaks, California USA; 2 Novartis Institutes for BioMedical Research, Cambridge, Massachusetts USA; 3 EMD Serono Research & Development Institute, Inc, Billerica, Massachusetts USA; 4 Biogen, Cambridge, Massachusetts USA; 5 Bill & Melinda Gates Medical Research Institute, One Kendall Square, Building 600, Suite 6-301, Cambridge, Massachusetts 02139 USA

**Keywords:** Anti-drug antibody, Bispecific therapeutic, Immunogenicity risk assessment, Multi-specific therapeutic, Oligomeric target

## Abstract

The objective of this manuscript is to provide the reader with a hypothetical case study to present an immunogenicity risk assessment for a multi-specific therapeutic as part of Investigational New Drug (IND) application. In order to provide context for the bioanalytical strategies used to support the multi-specific therapeutic presented herein, the introduction focuses on known immunogenicity risk factors. The subsequent hypothetical case study applies these principles to a specific example HC-12, based loosely on anti-TNFα and anti-IL-17A bispecific molecules previously in development, structured as an example immunogenicity risk assessment for submission to health authorities. The risk of higher incidence and safety impact of anti-drug antibodies (ADA) due to large protein complexes is explored in the context of multi-specificity and multi-valency of the therapeutic in combination with the oligomeric forms of the targets.

## INTRODUCTION

Immunogenicity risk assessment is an important component of biotherapeutic drug development, and part of the overall benefit risk assessment. A robust immunogenicity risk assessment process ensures that the most appropriate candidate molecules advance into the clinic, and that clinical immunogenicity is appropriately monitored. Risk assessment for multi-specific therapeutics can be especially challenging given that many molecules in this family can bind immune cells, trigger T cell activation, or bring together disparate molecules in a non-native way. Immunogenicity risk assessments include both the risk of generating an immune response and risk of clinical consequences if an immune response is generated. The latter is particularly critical to the overall benefit risk assessment and likely to inform the acceptable level of immunogenicity risk.

The potential clinical applications of multi-specific therapeutics are demonstrated by 3 approved products (Table I and II) and a breadth of molecules currently under investigation (1, 2). These include obligate concepts where having both specificities in the same molecule is critical (Table II), and combinatorial concepts where the two specificities do not necessarily need to be in the same molecule but may provide additional efficacy benefit over combination therapy (Table III). Of the 92 multi-specific therapeutics in clinical development as of March 2019 (2), 78 are for cancer and 14 in other indications such as autoimmune disorders, infectious diseases, hemophilia, diabetes, and ophthalmology. There are additional concepts that are no longer in development not represented in these tables, such as the anti-TNFα and anti-IL-17A bispecific hypothetical case study presented herein (3–7), and new concepts introduced since the source article was written in 2019 (2), such as the Ang-2 and VEGF bispecific faricimab (8).

**Table I:** FDA or EMA approved multi-specific therapeutics as of April 2021 including immunogenicity incidence reported on the label

Name	Company	Molecule description	Indication(s)	Immunogenicity (% Incidence)	Approval year
Removab® (9) (catumaxomab)	Fresenius Biotech and Trion Pharma Neovii Biotech GmbH	Rat-mouse hybrid IgG2 EpCAM, which is found in high levels on some types of cancer cells CD3, which is found on T cells	Malignant ascites	HAMA 94%	EMA 2009–2017^a^
Blincyto® (10, 11) (blinatumomab)	Amgen	CD19/CD3	Relapsed or refractory B cell precursor acute lymphoblastic leukemia (ALL) Acute lymphoblastic leukemia (ALL) who are in remission but still have minimal residual disease (MRD)	< 2%	FDA 2014
Hemlibra® (12, 13) (emicizumab)	Chugai and Roche	FIXa x FX	Routine prophylaxis of patients with hemophilia A with and without FVIII inhibitors	3.5% ADA < 1% nAb	FDA 2017

^a^Removab (catumaxomab) was previously approved by EMA with the subsequent removal at the request of the license holder

**Table II:** Obligate concept multi-specific therapeutics in clinical development as of March 2019

Mechanism of action	Targets	Indication
Bridging cells (in-trans): T cell redirection and/or activation	CD3 x B7-H3	Solid malignancies
	CD3 x BCMA	Hematological malignancies
	CD3 x CD123	Hematological malignancies
	CD3 x CD19	Hematological malignancies
	CD3 x CD20	Hematological malignancies
	CD3 x CD33	Hematological malignancies
	CD3 x CD38	Hematological malignancies
	CD3 x CEA	Solid malignancies
	CD3 x CLEC12A	Hematological malignancies
	CD3 x DLL3	Solid malignancies
	CD3 x EGFRvIII	Solid malignancies (EGFRvIII + glioblastoma)
	CD3 x EpCAM	Solid malignancies
	CD3 x FcRH5 (CD307)	Hematological malignancies
	CD3 x FLT3	Hematological malignancies
	CD3 x GPC3	Solid malignancies
	CD3 x gpA33	Solid malignancies
	CD3 x GPRC5D	Hematological malignancies
	CD3 x HER2	Solid malignancies (HER2 +)
	CD3 x MUC16	Solid malignancies
	CD3 x P-cadherin	Solid malignancies
	CD3 x PSMA	Solid malignancies (prostrate)
	CD3 x SSTR2	Solid malignancies
	CD3 x HIV-1 Env	HIV-1 infection
Bridging cells (in-trans): NK cell redirection and/or activation	CD16A x CD30	Hematological malignancies
	CD16 x CD33	Hematological malignancies
Bridging cells (in-trans): immune cell redirection and/or activation	CD40 x MSLN	Solid malignancies
	PD-L1 × 4-1BB	Hematological and solid malignancies
Bridging receptors (in-cis)	HER2 x HER3	Solid malignancies (breast cancer)
	EGFR x MET	Solid malignancies
	PD-1 × ICOS	Solid malignancies
	CD32b x CD79b	Immune-mediated disorders
	FGFR1 x KLB	Diabetes
Cofactor mimetic	FIXa x FX and/or FXa	Hemophilia A
Piggyback	Psl x PcrV	Pneumonia in mechanically ventilated subjects
Piggyback (bispecific molecules for half-life extension)	IL-6R x HSA	SLE and rheumatoid arthritis
	TNFα x HSA	Rheumatoid arthritis

Adapted from (2)

**Table III:** Combinatorial concept multi-specific therapeutics in clinical development as of March 2019

Mechanism of action	Targets	Indication
Targeting tumor heterogeneity	CD19 x CD22	Hematological malignancies
	EGFR x MET	Solid malignancies
	EGFR x LGR5	Solid malignancies
Targeting ligand redundancy	ANG2 x VEGF	Solid malignancies
	DLL4 x VEGF	Solid malignancies
	BAFF x B7RP1	SLE and rheumatoid arthritis
	BAFF x IL-17A	Sjögren syndrome
	IL-17 × IL-13	Asthma
	IL-23 × CGRP	Autoimmune diseases
	IL-4 × IL-13	Diffuse cutaneous systemic sclerosis
	NGF x TGF	Painful osteoarthritis of the knee and painful diabetic neuropathy
	VEGFA x ANG2	Neovascular wet age-related macular degeneration and diabetic macular oedema
Targeting multiple checkpoints	PD-1 × CTLA-4	Solid malignancies
	PD-1 × LAG3	Hematological and solid malignancies
	PD-1 × TIM3	Solid malignancies
	PD-L1 x CTLA-4	Hematological and solid malignancies
	PD-L1 x LAG3	Solid malignancies
	PD-L1 x TIM3	Solid malignancies
Targeting checkpoint and tumor antigens	PD-1 × undisclosed TAA	Solid malignancies
	CD47 x CD19	Hematological malignancies
Increasing avidity: biparatopic bispecific antibodies	HER2 x HER2	Solid malignancies (HER2 +)
	HER2 x HER2 ADC	Solid malignancies (HER2 +)

Adapted from (2)

As there are no formatting requirements around authoring an immunogenicity risk assessment, this article is part of a series designed to provide examples for consideration. The document structure of the immunogenicity risk assessment presented here is a result of input from the authors and their view of a fit for purpose risk assessment that may be submitted to health authorities. It does not reflect a structure endorsed by health authorities nor the experiences of any particular sponsor. This content is generally consistent with a truncated version of the integrated summary of immunogenicity structure option proposed by health authorities (14, 15), with removal of sections not pertinent depending on the development stage of the biotherapeutic program.

### Sequence Considerations

As with all protein therapeutics, one of the main drivers of risk of developing an immune response for a multi-specific therapeutic is the primary amino acid sequence and T cell epitope content (16). Any protein that has been engineered to change the amino acid sequence, has linker regions that introduce new linear epitopes that are not present in either parent, or that has non-natural or modified amino acids may have an elevated risk of developing an immune response. Most multi-specific therapeutics contain these elements, and consequently, these novel sequences should be evaluated for immunogenic risk using available tools such as in silico prediction, *in vitro* T cell assays, MHC binding assays, or ex vivo models (17).

Additionally, multi-specific therapeutics employ diverse protein engineering techniques such as quadroma, knobs-in-holes (KIH), CrossMAb, Triomab, strand-exchange engineered domain (SEED), cross-over dual variable (CODV), DART® (Dual-affinity Retargeting), TRIDENT®, dock-and-lock (DNL), BiTE® (Bispecific T cell Engager), bispecific killer engager (BiKE), trispecific killer engager (TriKE), multi-specific antibody-based therapeutics by cognate heterodimerization (MATCH), nanobody, diabody, diabody-Ig, etc. to bring together specific recombinant domains in a non-native way for simultaneous multi-target recognition (2, 18–25). The extensive protein engineering required to design and optimize such novel multi-specific therapeutics with mono- or multi-valency could inadvertently increase the product attribute-related immunogenic risk for these recombinant molecules.

### Critical Quality Attributes

Product critical quality attribute (CQA)-related risk is another driver that is typically considered in protein therapeutic immunogenicity risk assessment (14, 26–28), and multi-specific therapeutics are no exception. The CQA risk factors such as protein aggregates, posttranslational modifications, host cell- and process-related impurities (such as host-cell proteins and DNA, endotoxin, chromatography resin, contaminants, and degradants, etc.), formulation excipients and container closure, etc. are also relevant to multi-specific therapeutics.

Given the non-native arrangement of disparate functional domains in multi-specific molecules, there is potentially a higher immunogenicity risk related to aggregation. For all other CQAs, the potential for an impact on immunogenicity risk is similar for mono-specific and multi-specific molecules.

### Mechanism of Action—Immune Modulation

As the industry gains experience with immunomodulatory multi-specific therapeutics, pharmacological or mechanism of action (MoA)-based immunogenic risk factors are emerging as a crucial factor impacting the immune response to the drug. For instance, does the protein involve antagonism of more than one immune checkpoints, does it bind B cells, and/or does it agonize a costimulatory molecule? If the answer is yes to one or more of these possibilities, the molecule may have a higher than average risk of eliciting anti-drug antibodies (ADA), to be assessed during the clinical program. Data from combination studies with nivolumab and ipilimumab have clearly demonstrated this concept. When used as a monotherapy, nivolumab had a reported incidence of anti-nivolumab antibodies of 11.2%. As a combination therapy with ipilimumab, however, the incidence jumped to 37.8%, presumably based on the same antibody assay (29, 30). A similar finding was observed with durvalumab and tremelimumab (31). Multi-specific therapeutics that combine multiple immune stimulatory domains into the same molecule will carry this enhanced immunogenic risk.

When assessing MoA-based immunogenicity risk factors, direct binding to B cells of at least one domain of the multi-specific therapeutic must be considered. For example, a multi-specific therapeutic may be intended to deliver a signal to a specific subset of cells expressing target X via linkage of the signaling domain to an anti-target X antibody. In this scenario, B cell clones that recognize the antibody may also efficiently receive the signal. If the signaling domain has the ability to modulate B cell biology, then the antibody response to the therapeutic could be either enhanced or diminished.

Evidence also suggests that inclusion of a CD3 binding domain into a multi-specific therapeutic may be an immunogenicity risk factor. In general, molecules that bind CD3, such as otelixizumab or teplizumab, appear to be prone to ADA responses (32, 33) and this may hold true for T cell engagers as well, with AMG 211 eliciting antibodies in all subjects treated at > 3.2 mg in a phase 1 study (34). There are several plausible hypotheses for this finding depending on whether the mechanism is T cell engagement or T cell binding. For mechanisms engaging T cells, ADA responses may be more likely in the presence of wide-spread T cell activation. For T cell binders that are not intended to activate T cells, anti-CD3 domains may increase linkage of T cells to B cells that recognize the other domain(s) of the multi-specific therapeutic, potentially enhancing the likelihood of drug-specific B cell clones receiving T cell help.

On the other hand, the MoA of some biotherapeutics may also mitigate the risk of generating an immune response. One potential example of this is blinatumomab, which depletes CD19 + B cells. Even though it is composed of 2 murine single chain antibodies, the clinical immunogenicity incidence remains less than 2% (10, 11). Other multi-specific therapeutics may mitigate the immune response via other mechanisms, such as expanding regulatory T cells.

### Mechanism of Action—Complex Formation

Another MoA-based immunogenicity risk factor is the ability of the therapeutic to form large complexes with the target(s). This means targets that are oligomeric (as mentioned earlier) and either soluble or shed at significant levels pose the highest risk of large complex formation. Large complexes have long been hypothesized to be immunogenic (35, 36).

While there are no definitive examples of mechanistically proven, large complex-mediated immunogenicity to a therapeutic, strong correlative data exist. Adalimumab and infliximab both bind trimeric TNFα and form complexes of 4,000 kDa and 14,000 kDa, respectively; both are immunogenic in clinical studies, while etanercept does not form large complexes and is relatively non-immunogenic (37, 38). Multi-specific therapeutics can further enhance this complex forming ability since there are multiple targets. For instance, ABT-122 and JNJ-61178104 are both built on an adalimumab backbone and involve the addition of an IL-17A binding domain (39, 40). In healthy volunteer studies, ABT-122 and JNJ-61178104 have a reported ADA incidence of 99% and 100%, respectively, presumably due to enhanced ability to form large complexes (3, 41). In order to assess this risk, complex formation should be evaluated preclinically so that the ratio in which the therapeutic candidate binds the target is well understood. Any downstream effector function driven by the drug Fc domains should also be explored.

In addition to soluble complexes, multimeric membrane bound targets can mediate complex formation on the surface of cells. When these targets are expressed in antigen-presenting cells (APCs), it can facilitate complex uptake and increase the T cell mediated adaptive immune response (42).

### Treatment-Related Risk Factors—Patient and Regimen

As with all proteins, the intended patient population could also contribute to the immunogenic risk of multi-specific therapeutics. Important considerations include immune status of the patient population and prior exposure to related therapeutics. In an example for prior exposure, subjects with prior exposure to adalimumab have an increased immunogenic risk for a multi-specific therapeutic built on an adalimumab backbone. Immune status may be on a population level, such as autoimmune disease, or on an individual level, such as immunosuppressive methotrexate therapy.

Treatment-related immunogenicity risk factors such as administration route, dose, dosing frequency, treatment duration and concomitant immunomodulators (14, 27, 43, 44) are also critical elements in the immunogenicity risk assessment for multi-specific proteins. The extent of immune response due to treatment-related risk factors is dependent on the complex interplay between product-related intrinsic factors and patient-related extrinsic factors.

Historically, subcutaneous administration (SC) has been considered more likely to produce an immune response compared to intramuscular (IM) and intravenous (IV) routes; however, this has not been observed consistently for products with both SC and IV routes approved (45, 46). An analysis of clinical data for 27 non-oncology immunomodulatory antibodies showed no clinically meaningful difference in immunogenicity incidence between IV and SC administration routes (31). While the route of administration can be used to inform risk of positive seroconversion, it must be assessed in the clinic. SC administration may have an increased risk of protein aggregation due to dosing high concentrations in small volume (47). The aggregates and other antigenic attributes may cause an increase in the therapeutic protein uptake, processing and presentation by APCs resulting in immune responses (48). High therapeutic doses in some cases have been shown to saturate neutralizing Ab responses and restore therapeutic binding to its intended target. High doses may also induce long-term immune tolerance (49).

Longer treatment duration may also increase the risk of an immune response for protein therapeutics. The clinical immunogenicity incidence of IFNβ-1a-Rebif increased with a more frequent dosing regimen and subcutaneous administration relative to IFNβ-1a-Avonex (50, 51), with the caveat that this increase could be due to different study populations, immunogenicity assay formats, etc. While the highly potent immune-stimulatory multi-specific therapeutics may not require long treatment duration, the immune-suppressive multi-specific therapeutics may benefit from their pharmacological activity, irrespective of treatment duration. The concomitant medications (e.g., antihistamines, corticosteroids, methotrexate, interferons) and standard of care may also augment or reduce the antigen-processing of multi-specifics through their immunomodulatory effects.

### Potential Clinical Consequences

The risk of consequences from an immune response needs to be considered in addition to the risk of generating an immune response.

For multi-specifics that contain non-antibody components, a key aspect for multi-specific therapeutics is if one or more components resembles an endogenous counterpart, the potential consequences of cross-reactivity of ADA are determined by the function and uniqueness of that endogenous counterpart (52). Since the multi-specific therapeutic may be more immunogenic than the native protein, any increased incidence may adversely impact patient safety to a greater extent than occurs for the mono-specific compound.

While non-clinical studies are not predictive of the clinical incidence (16, 53, 54), they can provide valuable information on potential clinical consequences of an immune response if generated. For example, if a multi-specific therapeutic antibody, which was engineered to include an immunostimulatory cytokine, drives an enhanced immune response in cynomolgus macaques relative to the parent antibody alone, that enhancement may be clinically relevant if the cytokine biology is conserved across species. An important caveat to this nonclinical risk assessment approach is that immunogenicity driven by foreign sequence (in this case the human sequence) cannot be differentiated from MoA-driven immunogenicity with any degree of certainty and this must be acknowledged in the integrated immunogenicity risk assessment.

## HYPOTHETICAL CASE STUDY HC-12

**Fig. 1:**
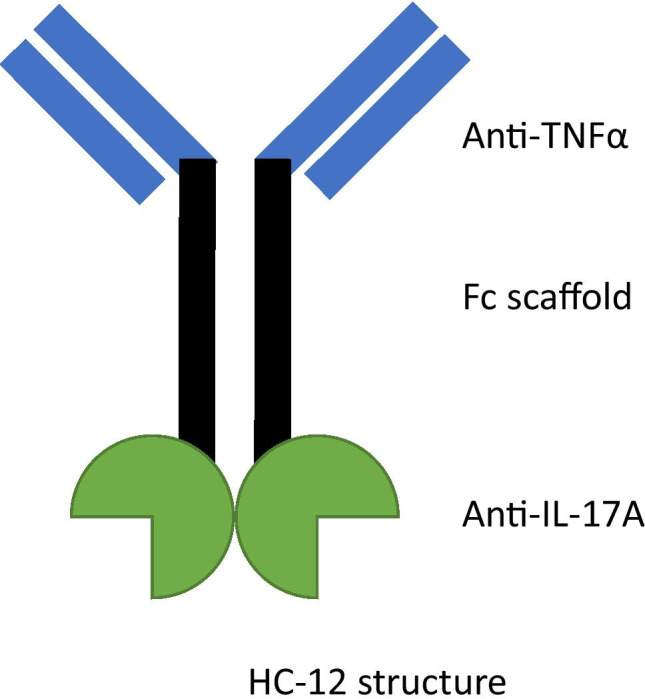
Structure of HC-12

In this hypothetical case study, HC-12 is based on a concept of an anti-TNFα monoclonal antibody (adalimumab) with anti-IL-17A domains fused to the C-terminus of the heavy chains (Fig. 1). Of the various TNFα and IL-17A bispecifics previously in development, it is closest to COVA-322/JNJ-63823539. Please note that none of the authors have worked on COVA-322/JNJ-63823539, and therefore, the contents of the actual immunogenicity risk assessment are unknown.

As illustrated below, the risk factors requiring the most attention vary depending on the molecule and intended use. The three areas of greatest attention for HC-12 were planned administration, primary sequence, and mechanism of action.

## IMMUNOGENICITY RISK ASSESSMENT

### Planned Administration

The planned administration is dosing SC every 3 weeks (q3w) for life-long treatment. With long-term exposure, seroconversion may occur at any point during treatment, so understanding the kinetics of ADA seroconversion during clinical trials will inform the cumulative risk of seroconversion for individual patients. This will help determine if there is a time period after which incidence has plateaued and patients who have not seroconverted are unlikely to do so.

For patients with autoimmune diseases on immunosuppressants, there would be gaps in treatment during life-long use. Patients are recommended to stop anti-TNFα immunomodulatory therapies for surgeries, during infections, and sometimes when receiving vaccinations (55, 56). If there are opportunities during the clinical development to evaluate rechallenge after these types of drug holidays, that could be helpful for prescribers to understand the potential impact of drug holidays on immunogenicity incidence of their patients. If opportunities arise during clinical development, such as gaps between a blinded study and an open-label extension or temporary cessation of treatment during rescreening for continued eligibility, immunogenicity samples will be acquired using the frequency at initial startup to evaluate potential accelerated kinetics of seroconversion. While gaps in treatment for immunosuppresants are hypothesized to potentially increase the risk of positive seroconversion *versus* continuous administration, there is a dearth of clinical data to test this hypothesis.

### Sequence-Based Risk

When bispecifics are generated, they may use fragments of IgG antibodies that are joined by a linker. The use of antibody fragments and the linker molecule may result in the generation of neo-epitopes that were not present in the full-length parental antibodies or uncover hidden (cryptic) epitopes that were buried in the 3D structure of the parental antibodies. Such epitopes may increase the immunogenicity risk for the bispecific compared to the parental antibodies.

To assess the sequence-based risk of HC-12, in silico analysis of MHC class II binding peptides (agretopes) was carried out using the Immune Epitope Database (free access at http://www.iedb.org, paid licenses for local installation behind a firewall). In silico analysis focused on the novel sequences from the candidate IL-17A binding domains and associated linkers which had not previously been administered to humans. An in silico score was calculated for each candidate molecule taking into account the strength and breadth of the predicted agretopes, and the IL-17A binding domain with the lowest in silico score was selected.

In silico analysis does not take into account TCR recognition; therefore, sequence-based risk was further assessed using an *in vitro* T cell assay (such as an ELISpot or flow cytometry assay). The parent monoclonal antibody adalimumab was compared to the full HC-12 molecule to assess the additional risk posed by the IL-17A binding domains and linker sequence. Both adalimumab and HC-12 elicited similar T cell responses in a panel of 50 donors, indicating that the addition of the IL-17A binding domains did not enhance sequence-based risk in naïve subjects.

### Mechanism of Action-Based Risk

Antagonism of two pro-inflammatory molecules, TNFα and IL-17A, is not expected to enhance immunogenicity and may cause a slight mitigation of immunogenicity by inhibiting inflammation. Consequently, there is a low risk of immunogenicity based on downstream target biology. However, both targets are oligomeric and soluble. TNFα is a homo-trimeric cytokine and IL-17A can exist in solution as a homo-dimer or a hetero-dimer with IL-17F.

Although TNFα is usually considered to be a soluble protein, it exists in a transmembrane form. It has been proposed that the high incidence of immunogenicity observed with anti-TNFα antibodies may be partially explained by transmembrane TNFα-mediated therapeutic uptake and antigen presentation on professional APCs (42).

Aggregates with highly repetitive structures can elicit ADA responses by directly activating B cells by the T cell independent mechanism (57). Both B1 and MZ B cells produce a rapid response that does not involve affinity maturation or the development of memory resulting in short-lived, low affinity and broad specificity. Furthermore, immune complexes can also trigger T-dependent antibody production (35) and the formation of antigen–antibody immune complexes is often used as a vaccination strategy.

**Fig. 2:**
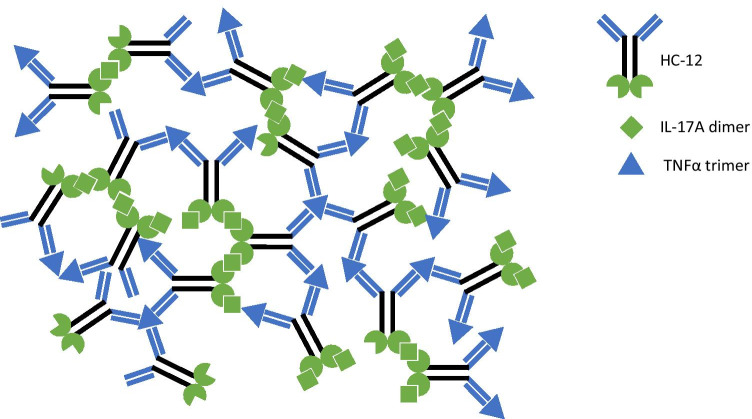
Potential quaternary structure of HC-12, TNFα, and IL-17A

HC-12 has 2 binding domains for each target, raising the possibility of significant protein complex formation (Fig. 2). In order to assess this risk, HC-12 was mixed at various ratios with TNFα and IL-17A and immune complex formation was assessed using SEC-MALS. At high drug to target ratios, immune complex formation was limited. When HC-12 and targets were present at roughly equimolar concentrations, large immune complexes of up to 6,000 kDa were observed.

While concerning, a similar phenomenon is observed with the HC-12 parent monoclonal antibody, adalimumab. Complexes of up to 4,000 kDa of adalimumab and TNFα were observed *in vitro* (38). The introduction of a second oligomeric target binding sequence into HC-12 could further enhance immunogenic risk relative to adalimumab, and the potential for immune complex formation contributes significantly to the immunogenic risk of HC-12.

### Attribute-Related Risk

HC-12 drug product attributes may impact immunogenic risk. The primary risks are formation of HC-12 aggregates and complementarity determining region tryptophan oxidation. Levels of these attributes in HC-12 drug substance are low and within target ranges, indicating that the attribute-related risk of immunogenicity is low.

### Excipient-Related Risk

The level of formulation excipients such as polysorbate 80 and sucrose in HC-12 are within target ranges and comply with United States Pharmacopeia-National Formulary, European Pharmacopoeia, and Japanese Pharmacopeia guidelines. Formulation components are not expected to impact immunogenic risk.

### Patient-Related Risk

HC-12 will be administered subcutaneously to subjects with autoimmune disease. In the absence of any immunosuppressive concomitant medications, the risk of immunogenicity in this population and with this route of administration is generally thought to be higher relative to administering a therapeutic intravenously to immunosuppressed oncology patients (45).

### Summary

The primary driver of immunogenic risk for HC-12 is pharmacological immunogenic risk; specifically, the formation of large protein complexes with target, particularly for this format with four binding moeities. Secondary to this risk is patient-related immunogenic risk due to the target population and route of administration. The clinical study will be designed with these risks in mind, and a dosing scheme has been selected which will maintain a high ratio of HC-12 to target in patient serum in order to reduce immune complex formation as much as possible. Based on this immunogenicity risk assessment, immunogenicity incidence will be carefully monitored in the first in human study, and a robust bioanalytical strategy has been devised.

## BIOANALYTICAL STRATEGY FOR HC-12

Successful implementation of the bioanalytical strategy ensures that scientific, data-driven decisions are made towards the clinical advancement of the therapeutic drug and in the context of the immunogenicity risk assessment. The strategy primarily involves the appropriate selection and development of bioanalytical assays measuring therapeutic drug concentrations and detection of ADA which may have developed during treatment. However, considering pharmacodynamic (PD) assessments as part of the bioanalytical strategy is also highly encouraged, as the utility of integrating pharmacokinetic (PK), ADA, and PD data may prove beneficial in understanding clinical impact of immunogenicity. As it pertains to the selection and development of the bioanalytical assays, it is important to apply scientific and strategic judgement, considering aspects related to assay format, pre-analytics of sample collection, as well as understanding the underlying biology of the target and the therapeutic.

### Pharmacokinetic Assays

For the case study of HC-12, measuring the clinically relevant therapeutic concentrations by means of a free PK assay format is recommended (58). A free assay format is designed to measure the drug that is unbound to targets and therefore available for binding. Due to the design, it is also the most sensitive to interference by antibodies binding the active sites, and therefore can suggest neutralizing activity of an ADA response when available PD and ADA data are integrated together (i.e. association of reduced PK and PD with ADA positivity) (58). The bispecific nature of HC-12 and circulating concentrations of oligomeric TNFα and IL-17A, make it an intricate process to select the most suitable free PK assay format (59). With a cellularly expressed target TNFα, it is not possible to do a total assay in serum since a proportion of antibody will be bound to the cellular portion. Please note that all free assay formats will underestimate the total protein concentration since intentionally measuring the portion that has binding arm(s) available. In addition, the free concentration will increase during the assay process as both dilution and exposure to reagents will alter the equilibrium.

A ligand-binding assay utilizing an anti-idiotype antibody to adalimumab, or immobilized TNFα, may be one approach to capture free HC-12 in the collected patient samples. Once HC-12 is captured, it can then be detected in the assay via an anti-human antibody conjugated to HRP or a reagent that binds anti-IL-17A. The former approach, however, will not distinguish whether IL-17A is bound, or not, to the IL-17A selective domains fused to the C-terminus of the antibody’s heavy chains. Instead, our recommendation is to pursue a PK assay format utilizing the unoccupied IL-17A selective domains as means of detecting the anti-adalimumab-captured HC-12 as the primary PK assay. To accomplish this, IL-17A or an antibody specific to the domains, which also blocks their binding to IL-17A, may be used. When using anti-idiotype reagents, binding surfaces may mimic conformational epitopes of targets. Therefore, target interference will be evaluated during reagent selection and assay development.

Please note that this intact free assay format can only detect molecules that have both an unoccupied anti-TNFα and an unoccupied anti-IL-17A. Since HC-12 is not an obligate bispecific concept, additional assays measuring free adalimumab and free anti-IL-17A may also be needed pairing the anti-idiotype reagents with anti-human immunoglobulin framework, with the understanding that there is overlap between the species detected in the three assays (59). In addition, a total serum assay may be needed to characterize the risk for complex formation, acknowledging that it is not a true total assay because the cell-bound portion is not detected.

### Immunogenicity

Similar to the multi-domain biotherapeutic-specific considerations for PK bioanalytical strategies, drug development-phase appropriate scientific and strategic considerations must be applied to the selection and development of immunogenicity assays for multi-specific therapeutics. For the case study of HC-12, a bridging assay format where study samples are incubated with therapeutic drug labeled with either capture (i.e., biotin) or detection (i.e., digoxin, ruthenium) molecules, was selected for detecting the presence of both low and high affinity antibodies against HC-12. Though this bridging assay format captures ADA of multiple binding affinities and isotypes, it does require that drug tolerance of the assay is appropriate for the anticipated samples to be tested, ensuring meaningful ADA detection even in those samples expected to contain high trough concentration (C_trough_) levels of therapeutic drug.

Related to the mechanism of action of HC-12, which targets the neutralization of TNFα and IL-17A cytokines, it is of great relevance to know that both TNFα and IL-17A exist as trimeric and dimeric proteins, respectively (60, 61). Due to the oligomeric nature of these targets, the presence of circulating TNFα and/or IL-17A in study samples could facilitate the target-mediated bridging between the labeled drugs intended to detect presence of ADA in the assay, therefore yielding a false positive result. Hence, during development of the ADA assay, it is important to understand if certain concentrations of oligomeric TNFα and/or IL-17A cytokines will allow bridging of the labeled drugs. This can be assessed by spiking in titrating concentrations of IL-17A and TNFα cytokines in the ADA assay, starting at 1,000-fold over the expected circulating endogenous concentration of each cytokine for the relevant disease indication. This excess is used to reflect possible increases in total target in the presence of binding antibodies (3, 58). Various pretreatment steps could be explored such as stripping excess target, longer incubation times in reagent excess, or switching to a sandwich format with anti-idiotype detection. At least one adalimumab biosimilar was able to successfully implement a bridging format even with the trimeric TNFα target (62).

Upon the successful testing of study samples, there may be incremental value to characterize if the ADA against HC-12 is targeted towards the therapeutics’ IL-17A selective domain or the adalimumab domain. One quick approach which may provide insight, would be to separately incubate confirmed ADA positive study samples with excess adalimumab lacking the IL-17A selective domain or with the IL-17A selective domain fused to another antibody of a similar isotype to adalimumab. To ensure ADA specificity is to the IL-17A selective domain for the latter, it would also be recommended to incubate study samples with the isotype control to adalimumab antibody lacking the IL-17A selective domain moiety. Testing these conditions in the ADA assay, coupled with a loss or reduction in signal, would provide valuable insight on the characterization of ADA against HC-12.

### Neutralizing Antibodies

While it is important to assess neutralizing anti-drug antibodies (nAb) throughout clinical development of HC-12, we propose to not develop a dedicated neutralizing antibody assay. Instead, we propose a data integration approach utilizing the available data from assays such as pharmacokinetic (free PK format), PD and ADA, as more informative than the use of a stand-alone nAb assay (63). For HC-12, a reduction of free drug concentration in ADA-positive samples would indicate reduced biological activity either directly via neutralizing antibodies, indirectly via clearing antibodies, or even partially bound species. With no endogenous counterparts to the two HC-12 moieties, there is not a mechanistic safety risk for nAb, so it may not be critical to differentiate between the possibilities. For a therapeutic with an antagonistic mechanism of action which is deemed low immunogenic risk, based on considerations such as the target, sequence homology to endogenous molecules and other factors discussed throughout, data from a standalone nAb assay would not provide additional insight into clinically meaningful neutralizing activity. Moreover, and where appropriate, recent guidance from FDA recognizes the potential of the data integration approach as a means of informing on the neutralizing activity of an anti-drug antibody response (64).

Lastly, the bioanalytical strategy of a therapeutic is predominantly comprised of the strategic selection and development of assays informing on therapeutic concentrations as well as presence of anti-therapeutic antibodies. Even though PD biomarkers are mainly driven by program and study specific aims, the apparent value of integrating PD data along with PK and ADA supports that when appropriate, PD assessments should also be taken into consideration as part of the overall bioanalytical strategy. Thus, biomarker and bioanalytical scientific leads need to work closely together and leverage synergies during the course of program’s development. For the purposes of the HC-12 case study, appropriate PD assessments, based on the therapeutics’ proposed mechanism of action, may include the quantification of circulating free TNFα and IL-17A cytokines as well as autoimmune disease markers. Though more challenging and complex, other PD assessments may include TNFα and/or IL-17A pathway-specific cellular responses on the cellular subset(s) of clinical interest for the indication. Ultimately, based on hypothesis, such PD biomarkers should track well with drug concentration levels and be sensitive enough to detect nAb responses.

### Sampling Schedule

Since the totality of data needs to be incorporated into the interpretation of the immunogenicity impact, sampling time points also impact interpretation. For HC-12, a more frequent sampling schedule is recommended for the evaluation of ADA with matched PK and PD analysis. To evaluate the kinetics of seroconversion in the phase 1 and phase 2 studies, sampling is recommended prior to dosing at day 1, at C_trough_ prior to subsequent doses every 3 weeks through month 6, and at C_trough_ prior to subsequent doses every 12 weeks thereafter. It is anticipated that more PK and PD samples will be needed for other development questions. In patient studies, monitoring is recommended for at least 24 months assuming that patients will be given the option to enroll in an open-label extension. Batch testing is recommended every 1–3 months as samples accumulate. The time points and testing frequency can be decreased for the phase 3 study pending seroconversion kinetics and incidence. While a transient peak of IgM development may be missed at 1–2 weeks, the 3-week interval aligned with trough concentrations increases the likelihood of detecting early seroconversion.

## DISCUSSION

The presented hypothetical case study illustrates the importance of understanding the potential quaternary structure of the drug and target that may lead to large structures. These structures can increase immunogenicity incidence by presenting as large immune complexes. In particular, the known immunogenic risk for adalimumab, hypothesized as a consequence of large complexes from multi-valency combined with oligomeric target, may have been further exacerbated by adding multi-valency to another oligomeric target in the multi-specific HC-12. While comparing immunogenicity incidence rates between molecules is fraught with assumptions, this comparison generates a viable hypothesis that should be considered in immunogenicity risk assessments of other multi-specific therapeutics. For visual scientists, drawing the potential complexes can be a useful tool (as shown in Fig. 2), understanding that there would be a distribution of sizes for any complex formation. If ADA are developed against the drug, these complexes can grow even larger leading the risk of safety sequelae related to immune complex deposition including glomerulonephritis (65).

Data from past clinical trials of TNFα and IL-17A multi-specific therapeutics illustrate how the risk assessment could have informed candidate selection and clinical development. As discussed above, the HC-12 matches the stoichiometry of COVA322/JNJ-63823539 (bivalent for both TNFα and IL-17A) while both ABT-122 and JNJ61178104 both bind only one TNFα and IL-17A molecule each (66, 67). In the FIH study for COVA322 (68), patients with stable chronic moderate-to-severe plaque psoriasis received ascending single-doses of COVA322 or placebo as a constant-rate IV infusion followed by 12 weeks of evaluation. The immunogenicity and pharmacokinetics have been published, and an immune response against either the adalimumab or fynomer moieties was observed with the ADA leading to an accelerated clearance of COVA322 compared to the parental molecules and ADA incidence of 93.3% (6, 69). The clinical trial was terminated in 2016 based upon the observed safety profile of COVA322 (68). While the safety results of this clinical trial have not been published as of writing this article, the safety findings were presumably not observed in the cynomolgus model with a NOAEL of 100 mg/kg/week, the highest dose tested (40). The ADA incidence was also high for JNJ61178104 (100%) and ABT-122 (99% healthy volunteers, 34% across autoimmune patients on stable methotrexate immunosuppression) (3, 41). For JNJ61178104, the immunogenicity was significantly higher than the two parent monospecific molecules golimumab (1.3%) and JNJ54160444 (0%), which presumably used comparable assays (3). These data, taken together, show a high risk of developing ADA for bispecific anti-TNFα/IL-17A antibodies. Both JNJ61178104 and ABT-122 were well-tolerated, with no significant safety findings (3, 41). ABT-122 was discontinued due to no efficacy improvement over the monospecific anti-TNFα (70). In the absence of published safety findings for COVA322, we hypothesize that larger immune complexes formed with the multivalent molecule could have been the differentiating factor between the bispecifics, all of which exhibited high immunogenicity. This further illustrates the importance of distinguishing between the risk of seroconversion and the risk of consequences from seroconversion.

## CONCLUSIONS

The vast majority of multi-specific therapeutics in the clinic are based on an immunoglobulin framework. As novel modalities emerge in the mono-specific space, it is reasonable to expect these to expand further into the multi-specific therapeutic space. The diverse immunogenicity risk assessments for those modalities may reveal additional risk factors impacting multi-specific therapeutics that are less prevalent on immunoglobulin scaffolds.

The valency of multi-specific therapeutics and targets can lead to large immune complexes *in vivo* per the presented hypothetical case study and published literature. The use of monovalent bispecific therapeutics may provide opportunities to target oligomeric targets with reduced immunogenic risk. Gathering additional information on the immunogenicity of bispecific therapeutics that reduce valency to oligomeric targets will further advance understanding of the impact of large complexes on immunogenic risk.

For multi-specific therapeutics in particular, it is critical that the potential impact of the pharmacology on immunogenicity is accounted for in the risk assessment. The combined impact of inhibiting or agonizing multiple immunomodulatory pathways can have a more significant impact on immunogenicity relative to what is observed with monospecific modalities.
